# The impact of macronutrient composition on metabolic regulation: An Islet‐Centric view

**DOI:** 10.1111/apha.13884

**Published:** 2022-09-12

**Authors:** Klinsmann Carolo dos Santos, Camilla Olofsson, João Paulo M. C. M. Cunha, Fiona Roberts, Sergiu‐Bogdan Catrina, Malin Fex, Neda Rajamand Ekberg, Peter Spégel

**Affiliations:** ^1^ Centre for Analysis and Synthesis, Department of Chemistry Lund University Lund Sweden; ^2^ Unit of Molecular Metabolism, Department of Clinical Sciences in Malmö Lund University Malmö Sweden; ^3^ Department of Molecular Medicine and Surgery, Karolinska University Hospital Karolinska Institute Stockholm Sweden; ^4^ Centrum for Diabetes Academic Specialist Centrum Stockholm Sweden

**Keywords:** alpha cell, beta cell, glucagon, insulin, insulin/glucagon ratio

## Abstract

**Aim:**

The influence of dietary carbohydrates and fats on weight gain is inconclusively understood. We studied the acute impact of these nutrients on the overall metabolic state utilizing the insulin:glucagon ratio (IGR).

**Methods:**

Following in vitro glucose and palmitate treatment, insulin and glucagon secretion from islets isolated from C57Bl/6J mice was measured. Our human in vivo study included 21 normoglycaemia (mean age 51.9 ± 16.5 years, BMI 23.9 ± 3.5 kg/m^2^, and HbA1c 36.9 ± 3.3 mmol/mol) and 20 type 2 diabetes (T2D) diagnosed individuals (duration 12 ± 7 years, mean age 63.6 ± 4.5 years, BMI 29.1 ± 2.4 kg/m^2^, and HbA1c 52.3 ± 9.5 mmol/mol). Individuals consumed a carbohydrate‐rich or fat‐rich meal (600 kcal) in a cross‐over design. Plasma insulin and glucagon levels were measured at −30, −5, and 0 min, and every 30 min until 240 min after meal ingestion.

**Results:**

The IGR measured from mouse islets was determined solely by glucose levels. The palmitate‐stimulated hormone secretion was largely glucose independent in the analysed mouse islets. The acute meal tolerance test demonstrated that insulin and glucagon secretion is dependent on glycaemic status and meal composition, whereas the IGR was dependent upon meal composition. The relative reduction in IGR elicited by the fat‐rich meal was more pronounced in obese individuals. This effect was blunted in T2D individuals with elevated HbA1c levels.

**Conclusion:**

The metabolic state in normoglycaemic individuals and T2D‐diagnosed individuals is regulated by glucose. We demonstrate that consumption of a low carbohydrate diet, eliciting a catabolic state, may be beneficial for weight loss, particularly in obese individuals.

## 1 INTRODUCTION

Glucose homeostasis is tightly controlled by pancreatic hormones. The anabolic hormone insulin is secreted from the islets of Langerhans when blood glucose is high, whereas the catabolic hormone glucagon is secreted from the islets when blood glucose is low. Insulin promotes glucose uptake and the storage of excess energy in the form of glycogen and triglycerides. Systemic hyperinsulinaemia (a hallmark of prediabetes), as a result of insulin hypersecretion from the beta‐cell, promotes weight gain due to the systemic anabolic functions of insulin. Of note, weight gain is a frequently occurring side‐effect of insulin therapy in diabetes mellitus likely also due to enhanced levels and anabolic activity of insulin.^1^ On the other hand, secreted glucagon liberates glucose and fatty acids from glycogen and triglyceride stores, respectively.^2^ As such, glucagon infusions were initially posited as a therapeutic anti‐obesity tool,^3^ although this is not practical due to significant health risks associated with glucagon‐mediated, enhanced release of fatty acids, hypoaminoacidaemia, uraemia, and muscle wasting.^2^ Type 2 diabetes (T2D) is often considered a disease of insulin deficiency/insulin resistance. However, accumulating evidence identifies T2D as a bi‐hormonal disease, with a loss of paracrine signals from β‐cells.^4^ This results in increased glucagon secretion, excessive glucagon secretion in response to protein, and a failure of glucagon inhibition in high glucose conditions.^2^ As such, monitoring of both insulin and glucagon is required to provide a comprehensive understanding of the metabolic state. The insulin:glucagon ratio (IGR), introduced by R.H. Unger in the 1970s,^5^ provides a powerful tool to adequately measure the metabolic state by overcoming the limitations of single hormone measurements. The IGR is currently used to delineate the effects of glucose‐lowering drugs.^6^ Drugs, such as sulfonylureas and insulin, elicit an increased IGR and may be beneficial in conditions whereby there is insufficient insulin secretion. Conversely, drugs that decrease IGR, such as metformin and sodium–glucose co‐transporter‐2 (SGLT2) inhibitors, may be beneficial in hyperinsulinaemia and insulin resistance.^6^ In line with this, sulfonylurea treatment is associated with weight gain and increased risk for hypoglycaemia, whereas metformin and SGLT2 inhibitors are associated with weight loss and low risk of hypoglycaemia.^6^

The mechanism underlying hormone secretion is well defined in the β‐cell^7^ but less well determined in the α‐ and δ‐cells of the pancreas. It is established that glucose stimulates insulin^7^ and somatostatin^8^ secretion, but inhibits glucagon secretion.^9^ Fatty acids (FAs) stimulate the secretion of glucagon and insulin,^7^ an effect that may depend on the level of glucose.^10^, ^11^ FAs have also been shown to inhibit somatostatin secretion.^8^ Additionally, glucagon secretion is inhibited by insulin and somatostatin,^8^ while insulin is stimulated by glucagon in a glucose‐dependent manner,^12^ but inhibited by somatostatin. Furthermore, somatostatin is stimulated by glucagon and may also be regulated by insulin.^8^, ^13^ Moreover, cell–cell interaction networks and neuronal control^14^ are important regulators in the control of insulin secretion.^15^ T2D has been linked with alterations in several of these regulatory mechanisms, including increased alpha‐cell insulin resistance,^16^ elevated circulating somatostatin levels,^17^ and at least in animal models, somatostatin hypersecretion.^8^

Concerted studies of insulin and glucagon are necessary to understand the effects of dietary modifications on obesity and diabetes.^18^ Studies have revealed hyperinsulinaemia to be an independent predictor of diabetes.^19^ In line with this, mouse models genetically modified to reduce insulin levels show resistance towards diet‐induced weight gain.^20^ Taken together, hypersecretion of insulin may be a trigger of obesity, thereby increasing the risk of developing T2D.^21^ However, without taking glucagon into account, implications of the overall metabolic state in diabetes risk will undoubtedly be overlooked.

In the present investigation, we examine the physiologically important, and largely undetermined to date, effects of FAs and glucose on the IGR in vitro. Next, we test whether our in vitro model can accurately predict the tight control of the metabolic state in vivo in response to variation in macronutrient composition and if this control is maintained in individuals diagnosed with T2D. Study participants with T2D were selected to represent a wide variation in HbA1c levels and the use of medication to better reflect the heterogeneity within the general population of individuals with T2D.

## 2 RESULTS

### 2.1 Alterations in glucose and palmitate levels and the insulin:glucagon ratio in vitro

**FIGURE 1:**
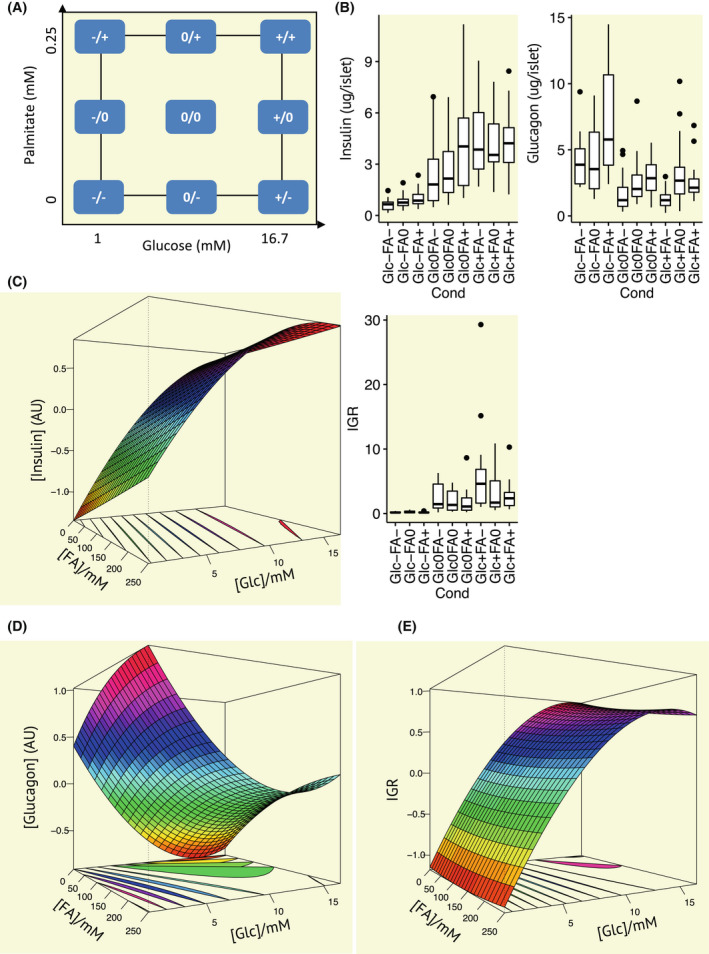
Alterations in insulin and glucagon secretion and the insulin:glucagon ratio (IGR) in islets from 10‐ to 16‐week‐old female mice elicited by glucose (Glc) and palmitate (FA) were examined according to a face‐centred central composite design. (A) Schematic representation of the face‐centred central composite design. Blue squares indicate the experiments that are conducted and include orthogonal experiments (corners), star‐point experiments (on the sides) and centre‐point experiments (centre) conducted in the experimental space, ranging from 0 to 0.25 mM palmitate and 1 to 16.7 mM glucose. Variables are scaled to +1 (16.7 mM glucose or 0.5 mM palmitate), −1 (1 mM glucose or 0.25 mM palmitate), and 0 (8.85 mM glucose or 0.125 mM palmitate). Hence, an experiment conducted with Glc‐ and Fa+ involves islets exposed to 1 mM glucose and 0.25 mM palmitate. (B) Glucagon and insulin secretion and the IGR for the experiments indicated in (A). Plots show the median, with boxes covering the first‐to‐third quartile, whiskers indicating 1.5 times the interquartile range, and dots show experimental results outside this range. Response surfaces for (C) insulin, (D) glucagon, and (E) the IGR derived from linear models (~Glc + FA + Glc*Glc + FA*FA + Glc*FA). Equations fitted from the data and used to produce the surface plots are given in Table 1

There is a lack of consensus regarding the impact of glucose and fatty acids on islet hormone secretion.^10^, ^11^ We, therefore, investigated the variation in insulin and glucagon secretion, and the IGR, over a wide range of glucose and fatty acid concentrations in islets from 10‐ to 16‐week‐old female mice. The fatty acid palmitate is the most dominant fatty acid in human plasma^22^ and so we utilized this in our models. Fatty acids, in general, have a stimulatory effect on insulin secretion.^11^ Our face‐centred central composite design allowed the examination of hormone secretion at all combinations of glucose and FA levels, ensuring analysis of all variations in interest. Importantly, this combination of factors at all possible levels allows further examination of the interactions between nutrients and non‐linear effects. Glucose and FA levels are expressed as high (+1), low (−1) or intermediate (0), ensuring that glucose will not dominate the model. This is critical given that glucose is found at higher physiological levels and varies in a larger concentration range than other nutrients. Experimental data were then fitted to glucose and palmitate levels using multilinear regression. The results, summarized in Table 1, show estimated slopes (effects) and significance for the model terms. These data are graphically represented (Figure 1B) and raw data are also presented (Figure 1C–E) as response surfaces derived from the model terms given (Table 1).

**TABLE 1:** Summary of models for insulin, glucagon and the insulin:glucagon ratio (IGR)

Response	G^a^	G*G^a^	P^a^	P*P^a^	G*P^a^	*R* ^2^	*R* ^2^_pred_
Insulin	12.6 (<2e^−16^)	−3.2 (3.7e^−7^)	4.3 (2.5e^−5^)	−0.030 (ns)	−0.00016 (0.011)	0.70	0.69
Glucagon	−7.3 (10e^−10^)	3.5 (6.6e^−6^)	3.4 (0.0054)	−1.1 (ns)	0.000085 (ns)	0.51	0.48
IGR	13.3 (<2e^−16^)	−4.0 (6.7e^−12^)	0.33 (ns)	0.31 (ns)	−0.00013 (0.022)	0.80	0.79

*Note*: *n* = 178 (*n* = 13–22 per condition), 184 (*n* = 17–24 per condition), and 158 (13–20 per condition) islet preparations from eight mice for insulin, glucagon, and IGR, respectively.

Abbreviations: G, effect of glucose; G*G and P*P, second‐order effect of glucose and palmitate, respectively; G*P, interaction between glucose and palmitate; ns, not significant; P, effect of palmitate; *R* ^2^, described variance; *R* ^2^_pred_, predictive *R* ^2^ by 10‐fold cross‐validation with three repeats.

^a^ Numbers indicate the effect (slope from the linear models) with the *p*‐value for the effect within brackets.

All calculated linear models were found to be valid (Table 1, Figure 1). Insulin secretion was stimulated by both glucose and palmitate levels. Glucagon secretion was stimulated by palmitate and inhibited by glucose. Notably, our model for insulin revealed only a very weak interaction between glucose and palmitate levels. As such, the stimulatory effect of palmitate on insulin secretion depends very little on glucose concentrations and vice versa. For glucagon secretion, this interaction was insignificant therefore suggesting that palmitate and glucose additively affect the hormone secretion. To produce a stronger depiction of glucose‐ and palmitate‐level variations on overall metabolic state regulation, we next examined the impact of these nutrients on the IGR. The IGR was calculated from each of the experiments individually. The resulting model revealed that glucose elicited a pronounced increase in the IGR, whereas palmitate was without effect on IGR. A very weak interaction between glucose and palmitate levels was observed utilizing this model.

### 2.2 Alterations in meal macronutrient composition and the insulin:glucagon ratio in vivo

**FIGURE 2:**
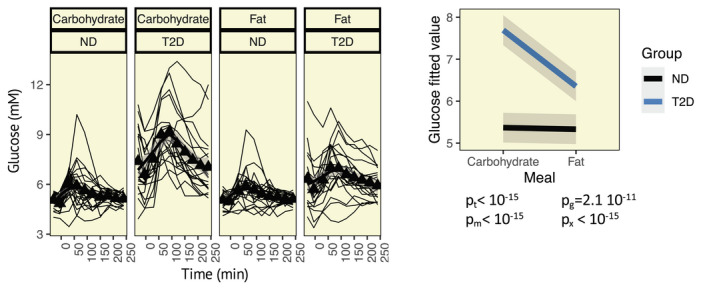
Meal‐elicited variation in blood glucose levels. Trajectories of glucose in individuals with type 2 diabetes (T2D) and without diabetes (ND) after consumption of a carbohydrate‐rich meal (carbohydrate) and a fat‐rich meal (fat). Thin black lines indicate trajectories for individual study participants, and black triangles and the thick black line indicate the mean and the grey shaded area the confidence interval. Right: Fitted values from linear mixed‐effects models. p‐values given below the graph; t, time; m, meal (carbohydrate or fat); g, group (T2D or ND); and x, interaction meal*group

**FIGURE 3:**
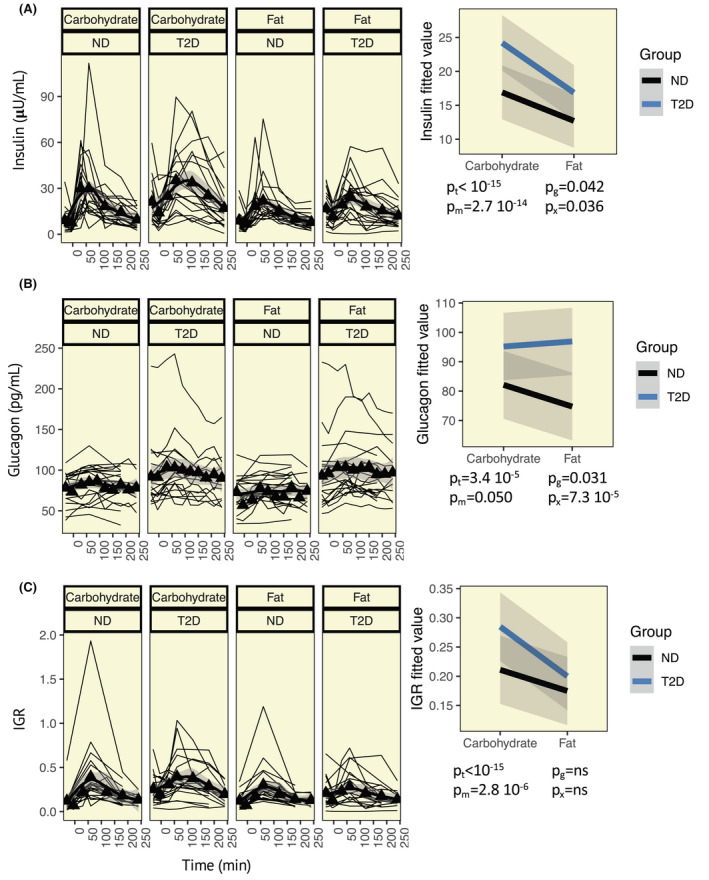
Meal‐elicited trajectories of hormones and the insulin:glucagon ratio (IGR). (A) Left: Trajectories of insulin in individuals with type 2 diabetes (T2D) and without diabetes (ND) after consumption of a carbohydrate‐rich meal (carbohydrate) and a fat‐rich meal (fat). Thin black lines indicate trajectories for individual study participants, black triangles and the thick black line indicate the mean, and grey shaded area the confidence interval. Right: Fitted values from linear mixed‐effects models. *p*‐values given below the graph; t, time; m, meal (carbohydrate or fat); g, group (T2D or ND); x, interaction meal*group. (B) Data for glucagon and (C) data for the IGR, illustrated as outlined in (A)

The IGR is known to differ between normoglycaemic individuals and individuals diagnosed with T2D^5^ and may further be impacted by gut‐derived physiological processes.^23^ Hence, after establishing that glucose is the main determinant of the IGR in isolated rodent islets, we examined whether similar effects are observed in humans in vivo and if the regulation of IGR is conserved in subjects with T2D. Therefore, we examined the dynamic response in insulin and glucagon levels, and the IGR after ingestion of one carbohydrate‐ and one fat‐rich meal (Table 2). In those with T2D, elevation of blood glucose levels was more pronounced following ingestion of the carbohydrate‐rich meal compared to the fat‐rich meal, whereas no difference was observed in normoglycaemic individuals (Figure 2). One individual in the T2D group showed extreme hyperinsulinaemia throughout both meal tests (insulin >400 mU/L) and was consequently excluded from the analyses. Insulin levels were significantly higher in participants with T2D, as compared to normoglycaemic individuals, reflecting the well‐known difference in basal insulin levels between these groups, and were higher in response to the carbohydrate‐rich meal, as compared to the fat‐rich meal (Figure 3A). The glycaemic state also impacted on the meal‐elicited insulin response, as indicated by a significant meal*group interaction. Similar effects were observed for glucagon (Figure 3B), where both the glycaemic state and the meal composition, as well as their interaction, showed significant effects on glucagon levels. Changes in the IGR depend upon the carbohydrate and fat content of the meal and this effect does not differ between those with and without T2D (Figure 3C). Notably, the exclusion of individuals taking GLP‐1 analogues and DPP4 inhibitors did not significantly alter these findings. To confirm that additional interactions are not causing biases in our analyses, we also included interactions with the time variable. The meal (*p* = 2.8e^−6^) and time (*p* < 10e^−15^) variables, but no additional variables or interactions, significantly influenced the IGR. Results also remained similar for glucagon when assessing analysis bias (time, *p* = 4.6e^−5^; group, *p* = 0.031; meal, *p* = 0.053; group*meal, *p* = 7.4e^−5^). Significant time interactions were observed for insulin (time*group, *p* = 0.00018; time*meal, *p* = 0.0038), in addition to significant effects of time (*p* < 10^−15^), group (*p* = 0.042), meal (*p* = 2.83e^−15^), and the interaction group*meal (*p* = 0.030).

**TABLE 2:** Macro‐ and micronutrient composition of the meals

Composition	Carbohydrate meal	Fat meal
Carbohydrate (%E)	52	32
Protein (%E)	19	18
Fat (%E)	30	50
Saturated (%E)	10	13
Monosaturated (%E)	11	28
Polyunsaturated (%E)	5	6
Fibre (g)	11	8
Ascorbic acid, mg	122	48
Tocopherols, mg	3	5
Carotenoids, μg	3866	2940
Riboflavin, mg	0.6	0.3
Selenium, μg	9	10
Zinc, mg	5	6

Next, we adjusted the models for potential confounders. After adjustment for BMI, the insulin response was dependent on the meal composition (*p* = 4.4e^−14^), time (*p* < 2.2e^−16^), BMI (*p* = 0.050), and the interaction between the meal composition and group (*p* = 0.040). The glucagon response was unaffected by BMI, depending only on time (*p* = 5.2e^−5^), meal composition (*p* = 0.045), group (*p* = 0.030), and the interaction between the two latter covariates (*p* = 7.0e^−5^). Finally, the IGR was associated with meal type (*p* = 3.8e^−6^), time (*p* < 2.2e^−16^), and BMI (*p* = 0.0033) but was independent of the glycaemic state.

Subsequently, we examined if diabetes duration, HbA1c, and the type of antidiabetic treatment impacted the meal response in participants diagnosed with T2D. Insulin levels decreased in those with a longer diabetes duration (*p* = 0.035), which translated into a similar reduction in the IGR (*p* = 0.019). The IGR also decreased with increasing HbA1c, which was possibly driven by a trend towards increased glucagon levels (*p* = 0.078), and a non‐significant increase in insulin was observed (*p* = 0.26). The use of DPP4 inhibitors was associated with a reduced IGR (*p* = 0.045) and GLP1 with reduced glucagon (*p* = 0.035). Insulin levels were higher in those on metformin (*p* = 0.048). In the non‐diabetic participants, HbA1c was associated with an increase in glucagon concentrations (*p* = 0.019), which translated into a lower IGR (*p* = 0.042). Meal composition and time remained significant in participants with diabetes (*p* = 6.5e^−7^ and 2.6e^−10^, respectively) and in normoglycaemic participants (*p* = 6.5e^−7^ and 1.7e^−10^, respectively).

### 2.3 Alterations in meal macronutrient composition and the proinsulin:insulin ratio in vivo

T2D is associated with an increased secretion of proinsulin from the β‐cells both in the fasted and the postprandial state.^24^ To examine whether insulin processing was affected by the glycaemic state and the meal composition, we investigated the proinsulin:insulin ratio. This ratio varied with time during the meal test (*p* < 10^−15^) but was uninfluenced by both the meal type and the glycaemic state of the test subjects.

### 2.4 Impact of BMI and glycaemic control on the meal‐elicited IGR response

High‐fat diets have been shown to induce a greater weight loss in obese and insulin‐resistant individuals compared to those that are leaner and less insulin resistant.^25^ Hence, we set out to examine whether the meal‐elicited alterations in the IGR (ΔIGR) (defined as IGR at 60 min and IGR at −30 min [30 min before meal intake; baseline]) were associated with BMI and HbA1c.

**FIGURE 4:**
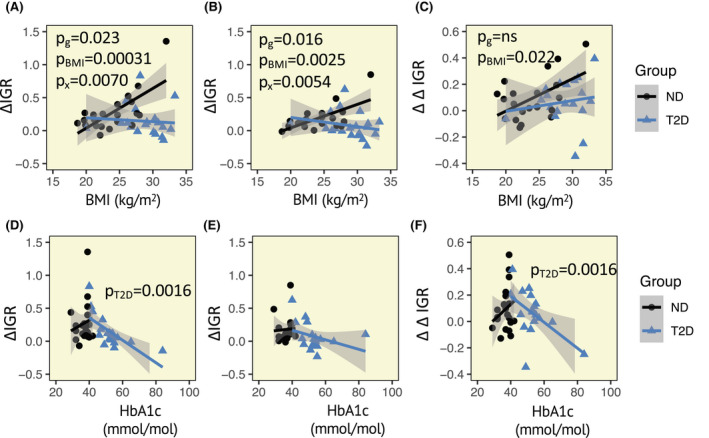
Influence of HbA1c and BMI on the meal elicited response in insulin:glucagon ratio (IGR). The meal‐elicited increase in IGR is substantially higher for both a carbohydrate‐rich meal (A) and a fat‐rich meal (B) in normoglycaemic obese individuals as compared to leaner individuals. No such association is observed for individuals with type 2 diabetes (T2D). (C) The relative increase in the IGR response for a carbohydrate‐rich meal as compared to a fat‐rich meal depends on the BMI, but not on the glycaemic state of the individual. The meal elicited response in the IGR depends on the HbA1c in individuals with T2D for the carbohydrate‐rich meal (D), but not for the fat‐rich meal (E). The relative increase in the IGR response for a carbohydrate‐rich meal decreases with HbA1c among individuals diagnosed with T2D. ΔIGR = IGR at 60 min – IGR at baseline (−30 min). ΔΔIGR = ΔIGR for carbohydrate‐rich meal – ΔIGR for fat‐rich meal. Data analysed using multilinear regression. *p*‐values given above the graph; BMI; g, group (T2D or ND); x, interaction BMI*group

First, we examined the influence of BMI on the IGR response. ΔIGR depended on the glycaemic status and the BMI for both meal types. Notably, ΔIGR showed a striking BMI dependence in normoglycaemic individuals but not in individuals diagnosed with T2D for meal types (Figure 4A,B). Finally, we assessed whether the relative lowering of the IGR in response to the fat‐rich meal depend on the BMI of the individual, by calculating the ΔΔIGR, defined as ΔIGR for the carbohydrate‐rich meal – ΔIGR for the fat‐rich meal. Notably, lowering of the IGR response by the fat‐rich meal was more enhanced in those with a higher BMI and there was no difference between individuals with or without T2D (Figure 4C). These results remained constant following the exclusion of individuals taking GLP‐1 analogues and DPP4 inhibitors.

Next, we examined ΔIGR and ΔΔIGR independently in participants with T2D and without T2D to allow for further examination of possible influence from HbA1c, diabetes duration, and medication. Among the participants diagnosed with T2D, ΔIGR for the carbohydrate‐rich meal was independent of BMI, diabetes duration, and medication, but showed a negative association with HbA1c (*p* = 0.00156) (Figure 4D). ΔIGR for the lipid‐rich meal was unaffected by all investigated covariates (Figure 4E). As a result, the ΔΔIGR showed a negative association with HbA1c (*p* = 0.012), showing a relative elevation in the carbohydrate‐rich meal for individuals with HbA1c >59.3 (Figure 4F). Again, results remained unaltered after exclusion of the individuals taking GLP‐1 analogues and DPP4 inhibitors. Among the normoglycaemic individuals, ΔIGR in response to both the fat‐ and carbohydrate‐rich meals depended on BMI (*p* = 0.00053 and *p* = 0.0013, respectively), but was independent of HbA1c. The ΔΔIGR was associated with BMI (*p* = 0.016).

## 3 DISCUSSION

A substantial number of studies examining nutrient‐elicited secretion of glucagon and insulin from islet cells have been published.^7^, ^26^ However, very little is known about the relationship between nutrients and the overall metabolic state, which is dictated by the concerted action of insulin and glucagon. Here, we conducted a systematic investigation on the impact of two major nutrients: glucose and the fatty acid palmitate, on IGR, serving as a proxy for global metabolic regulation.

Our in vitro data confirm that glucose stimulates the secretion of insulin and inhibits the secretion of glucagon, whereas palmitate stimulates the secretion of both hormones. There are conflicting data in the literature relating to glucose dependency on the stimulatory effect of fatty acids on pancreatic hormone secretion.^10^, ^11^ Our data reveal a significant, although very weak, interaction between glucose and palmitate levels in all models utilized. Hence, from a clinical viewpoint, palmitate largely elicits a glucose‐independent stimulatory effect on both insulin and glucagon secretion. The key finding in the present study is that the stimulatory effects of palmitate on secretion of both the anabolic hormone insulin and the catabolic hormone glucagon translates into glucose being the sole determinant of the IGR and the overall metabolic state.

Next, we aimed to translate these in vitro findings into a human in vivo study. Based on our in vitro model, we hypothesized that the IGR would be more responsive to a carbohydrate‐rich meal as compared to a fat‐rich meal. Moreover, we aimed to test whether the effects of these macronutrients on the IGR depended on the individuals' glycaemic state. In strong agreement with the in vitro data, we observed that the IGR was more robustly increased in response to a carbohydrate‐rich meal as compared to a fat‐rich meal. Hence, despite the fatty acid composition being inherently more complex in the meals, the results qualitatively recapitulate our findings from islets stimulated with the single fatty acid palmitate. Our results show that insulin and glucagon secretion are dependent on glycaemic state, in agreement with the hormonal dysregulation associated with T2D, whereas changes in the IGR were independent of glycaemic state. Although responses were dependent on BMI, HbA1c, diabetes duration, and medication, our analyses still showed that a carbohydrate‐rich meal elicits an anabolic state in both normoglycaemic individuals and those with T2D, at least in the short timeframe of this study. Notably, we could also show that the use of DPP4 inhibitors was associated with a lower IGR and that metformin use was more prevalent in those with high insulin levels, indicative of insulin resistance.

The present study focuses on the hormonal regulation of the metabolic state and does not take into account the nutritional impact on reward systems, satiety, and behaviour, which all play important roles in the regulation of body weight.^27^ Insulin is, however, known to promote hyperphagia, whereas glucagon may elicit the opposite effect.^28^ Utilizing the IGR likely provides better information on hunger compared to methods which assess the hormones separately. Still, other hormones, such as GLP‐1 and its analogues, which generally increase the IGR,^6^ also produce significant weight loss, via a complex influence on food intake.^29^ Hyperphagia alone does not explain all weight gain produced by elevated insulin levels,^30^ but may still contribute to the increased risk of obesity and T2D elicited by insulin hypersecretion.^19^, ^21^

Whether the acute relative increase in the IGR elicited by meals rich in carbohydrates translates into more long‐term effects on weight gain remains undetermined. However, the development and implementation of guidelines promoting a decreased fat intake during recent decades parallel the rise in obesity and diabetes prevalence. Still, a casual relation between meal macronutrient composition and weight loss has been difficult to establish,^31^ most likely due to the difficulty in designing and controlling such studies. Nevertheless, our findings of a reduced IGR in the low‐carbohydrate meal‐fed individuals support previous results demonstrating an increased energy expenditure associated with such diet types.^32^

A notable finding in the present study is the enhanced reduction in the IGR response with the high‐fat meal in obese individuals. The degree of obesity seems to impact the responsiveness to dietary modifications. Overall, very few studies on the effect of macronutrient composition on weight loss have stratified individuals based on obesity and or glycaemic state, which may explain some of the discrepancies in the literature.^31^ However, our findings are in line with a previous study demonstrating that obese and insulin‐resistant individuals lose more weight in response to high‐fat diet as compared to leaner, insulin‐sensitive individuals.^25^ A reduction in the postprandial IGR may explain, at least partially, this long‐term weight loss. Moreover, we find that the relative reduction in IGR elicited by a fat‐rich meal is only observed in those with an HbA1c below 59.3. Therefore, fat‐rich diets may not be efficient in eliciting a catabolic state in individuals with poor glycaemic control.

Further studies are necessary to assess the role of amino acids, reflecting the third major macronutrient, in the regulation of metabolic state. This represents a logistical challenge to the scientific community, requiring a substantial number of experiments to account for the differential impact of, and interactions between, different amino acids. Recent studies show that the amino acid composition of casein and soy elicits an increase and a decrease, respectively, in the IGR.^33^

A limitation of the present study is that the in vitro experiments were completed utilizing only islets of female mice of a limited age span. As such, age‐dependent effects and sexual dimorphism remain unexplored in the current study. By performing experiments in vitro, we decreased the potential impact of circulating sex hormones in our findings, but we acknowledge that potential influences of sex hormone receptors remain and should be further investigated. In our human studies, findings were replicated in both sexes. Another limitation of the study is the lack of data on incretin levels. Secretion of incretins varies in response to nutrients type and subsequently impacts downstream insulin and glucagon secretion. However, the incretin effect is most likely more quantitative than qualitative in nature, given that the macronutrient‐elicited regulation of the IGR was similar in vitro and in vivo. Moreover, as data on whole‐body metabolism are lacking, we are unable to describe potential effects resulting from thermic effects, which may differ between carbohydrate‐ and fat‐rich meals.^34^ Finally, experiments were not designed to evaluate the possible effects of somatostatin, which is linked with glucagon secretion in a reciprocal feedback cycle and represents a key regulator of insulin secretion.^35^

In conclusion, in our systematically designed attempt to bring dietary studies back to the islet and hormonal control, we show that rodent islets may serve as a relevant model to study nutrient‐elicited regulation of metabolic control in humans. Moreover, we find clear support for glucose as the main regulator of the metabolic state in both normoglycaemic individuals and individuals with T2D. Finally, we show that a low‐carbohydrate meal elicits a more pronounced decrease in the IGR in individuals with a higher BMI, independent of their glycaemic state, supporting the use of low‐carbohydrate meals to elicit a catabolic state which may be beneficial in eliciting weight loss in obese individuals with T2D.

## 4 MATERIALS AND METHODS

### 4.1 In vitro islet hormone secretion

Islets were isolated from 10‐ to 16‐week‐old female C57Bl/6J mice using collagenase digestion and handpicked under a stereomicroscope. Islets were pre‐incubated in a 48‐well plate (*n* = 5 islets per well) in HEPES‐buffered saline solution (HBSS; 114 mM NaCl, 4.7 mM KCl, 1.2 mM KH2PO4, 1.16 mM MgSO4, 20 mM HEPES, 2.5 mM CaCl2, 25.5 mM NaHCO3 and 0.2% fatty acid‐free bovine serum albumin, pH 7.2) supplemented with 2.8 mm glucose for 1 h at 37°C. Next, the buffer was exchanged for HBSS supplemented with 0–0.25 mM BSA complex bound palmitate produced from a stock solution containing 10% BSA and 0.1 mM palmitate^36^ and 1–16.7 mM glucose according to a face‐centred central composite design (Figure 1A) and incubated for another 1 h at 37°C. The palmitate/BSA stock solution contained 5% BSA and 0.5 mM palmitate, yielding a palmitate/BSA molar ratio of 1.7/1, which is in the range expected in a healthy human.^37^ The design investigates all factors at three levels, thereby allowing for the examination of linear and quadratic single‐factor effects and two‐factor interactions with a minimal number of experiments. Finally, the supernatant was collected, centrifuged, and assayed for secreted insulin (Mercodia Mouse Insulin ELISA 10–1247‐10) and glucagon (Mercodia Glucagon ELISA 10‐1271‐01). Islets isolated from 8 mice were divided into nine conditions, yielding 2–3 replicates per mouse (5 islets per experiment) and a total of 20–24 experiments per condition. Experiments yielding saturated ELISA signals were excluded (20 for insulin and 26 for glucagon) to avoid potential contributions from dying cells. National and institutional guidelines for the care and use of animals in this study were followed. All animal procedures were approved by the Malmö/Lund Committee for Animal Experiment Ethics, Lund, Sweden.

### 4.2 Study population

Study participants were diagnosed with T2D (*n* = 21) or without diabetes (non‐diabetic, ND, *n* = 21) (Table 3). Inclusion criteria were 20 > age > 75 years, 25 < BMI < 33 kg/m^2^, and disease duration >5 years. Exclusion criteria were heart or renal failure, liver disease, and treatment with pioglitazone. Participants were recruited at the Department of Endocrinology, Metabolism and Diabetes at Karolinska University Hospital or through advertisements. Subjects with T2D were treated with metformin (*n* = 15), with some individuals also receiving additional hypoglycaemic drugs (insulin, *n* = 5; DPP4 inhibitors, *n* = 3; sulphonylurea, *n* = 3; acarbose, *n* = 1; and GLP‐1 analogues, *n* = 2) (Table 3). Subjects treated with insulin secretagogues, including GLP‐1, were instructed to refrain from this medication the night before, or in the morning before, the intervention. DPP‐4 inhibitors were not discontinued, as they are not directly modulating insulin secretion. The study protocol was approved by the Regional Ethical Review Board in Stockholm (ClinicalTrial.gov Identifier: NCT02544568) and all participants signed a written informed consent.

**TABLE 3:** Subject characteristics. Values are median (min, max)

	T2D *n* = 21	ND *n* = 21	*p*
Age (years)	64 (55, 74)	57 (20, 74)	0.02
Male/female (*n*)	10/11	9/12	1.00
BMI (kg/m^2^)	29 (25, 33)	24 (19, 32)	<0.001
HbA1c (mmol/mol)	52 (40, 84)	37 (29, 42)	<0.001
Diabetes duration (years)	9 (5, 26)	‐	‐
No treatment (*n*)	5		
Metformin (*n*)	4		
+insulin (*n*)	3		
+GLP‐1 (*n*)	2		
+DPP4i (*n*)	3		
+sulphonylurea (*n*)	2		
+acarbose (*n*)	1		

### 4.3 Meal tolerance test

Study participants received two isocaloric meals (600 kcal), being either a high‐carbohydrate or low‐carbohydrate/high‐fat meal^38^ (Table 2) in a cross‐over design with at least 1‐ and 2‐month wash‐out period for men and women, respectively. The meals consisted of red meat, potatoes (boiled or French fries), and different vegetables/legumes, depending on meal compositions, and water to drink. The meals were prepared and served at a restaurant located at the Karolinska University Hospital. Study participants were unaware of meal composition and a research nurse oversaw the intake. Approximately 4 hours before the intervention, participants consumed a standardized breakfast at home. The breakfast constituted 400–450 kcal, with 56–66 energy per cent (%E) carbohydrate (8–10 grams of fibre), 21%–24%E protein, and 13–20%E fat. Blood samples were collected 30 and 5 min before the meal intake and then every 30 min from 30 to 240 min after meal intake, enabling sampling over the timeframe incorporating the largest variation in plasma glucose and lipid levels.^39^ Insulin was measured using ELISA (DAKO, Agilent Technologies), glucagon using RIA (GL‐32K; Millipore), and proinsulin using ELISA (10‐1118‐01, Mercodia).

### 4.4 Statistical analyses

All analyses were performed in R (version 3.6.1). Normality was tested using the Shapiro–Wilk test (shapiro.test, stats package) and non‐normal distributed variables log2‐transformed. In vitro data were evaluated using glucose and palmitate levels, set as −1, 0, or 1, and mouse identity as independent factors, and the levels of insulin and glucagon secretion and the IGR as dependent variables using multilinear regression (lm, anova, and stats package). An interaction between glucose and palmitate and quadratic terms for glucose and palmitate was included. Outliers were identified by the Cook's distance (cooks. distance, and stats package). Models were evaluated by cross‐validation (trainControl and caret package) and visualized as contour plots (rsm, rsm package and persp, and graphics package). In vivo data were evaluated using linear mixed‐effects models (lmer and lme4 package) and ANOVA (car package), with time, meal type, and glycaemic status (ND or T2D) and relevant interactions as fixed effects and the individual as a random effect. Predicted values were extracted from the linear mixed‐effects models using Effect (effects package). Associations between IGR and BMI were examined using multilinear regression. Data were visualized using ggplot (ggplot2 package).

## FUNDING INFORMATION

This work was supported by the Albert Påhlsson Foundation, the Crafoord Foundation, the Novo Nordisk Foundation, the Swedish Diabetes Foundation, the Hjelt Foundation, the Per and Ulla Schyberg Foundation, Dr P Håkansson's Foundation, Eslöv, Sweden, the Horizon 2020 Program (T2Dsystems), the Swedish Research Council (Linnaeus grant, Dnr 349‐2006‐237, and Strategic Research Area Exodiab, Dnr 2009‐1039), and the Swedish Foundation for Strategic Research (Dnr IRC15‐0067). Grants from Family Erling Persson and Ronald Adolfsson and Clinical Research Grant at Karolinska University Hospital, Strategic Research Program Diabetes, Bert von Kantzows Foundation, Swedish Society of Medicine, and Kung Gustaf V:soch Drottning Victorias Frimurarestifelse.

## CONFLICT OF INTEREST

The authors have no conflict of interest to declare.
